# Exploring the Link between Nucleosome Occupancy and DNA Methylation

**DOI:** 10.3389/fgene.2017.00232

**Published:** 2018-01-12

**Authors:** Cecilia Lövkvist, Kim Sneppen, Jan O. Haerter

**Affiliations:** Center for Models of Life, Niels Bohr Institue, University of Copenhagen, Copenhagen, Denmark

**Keywords:** CpG sites, DNA methylation, nucleosome occupancy, epigenetics, gene expression

## Abstract

Near promoters, both nucleosomes and CpG sites form characteristic spatial patterns. Previously, nucleosome depleted regions were observed upstream of transcription start sites and nucleosome occupancy was reported to correlate both with CpG density and the level of CpG methylation. Several studies imply a causal link where CpG methylation might induce nucleosome formation, whereas others argue the opposite, i.e., that nucleosome occupancy might influence CpG methylation. Correlations are indeed evident between nucleosomes, CpG density and CpG methylation—at least near promoter sites. It is however less established whether there is an immediate causal relation between nucleosome occupancy and the presence of CpG sites—or if nucleosome occupancy could be influenced by other factors. In this work, we test for such causality in human genomes by analyzing the three quantities both near and away from promoter sites. For data from the human genome we compare promoter regions with given CpG densities with genomic regions without promoters but of similar CpG densities. We find the observed correlation between nucleosome occupancy and CpG density, respectively CpG methylation, to be specific to promoter regions. In other regions along the genome nucleosome occupancy is statistically independent of the positioning of CpGs or their methylation levels. Anti-correlation between CpG density and methylation level is however similarly strong in both regions. On promoters, nucleosome occupancy is more strongly affected by the level of gene expression than CpG density or CpG methylation—calling into question any direct causal relation between nucleosome occupancy and CpG organization. Rather, our results suggest that for organisms with cytosine methylation nucleosome occupancy might be primarily linked to gene expression, with no strong impact on methylation.

## Introduction

In eukaryotes, a large number of epigenetic mechanisms are involved in packaging the genome in different chromatin states. These chromatin states provide information about gene function and cellular state (Bernstein et al., 2007; Mikkelsen et al., 2007). Two particular mechanisms, nucleosome occupancy and DNA methylation, have been reported to form specific patterns around promoters and these patterns were shown to correlate with gene expression (Portela and Esteller, 2010). With the existence of such correlations, it is investigated whether DNA methylation and nucleosome occupancy play a direct and decisive role in regulating gene activity (Vojta et al., 2016; Lai and Pugh, 2017; Rudnizky et al., 2017).

DNA methylation in eukaryotes mainly concerns CpG sites (Jaenisch and Bird, 2003; Antequera, 2007; Portela and Esteller, 2010), which are predominantly methylated and sparsely distributed over the genome (Gardiner-Garden and Frommer, 1987; Larsen et al., 1992; Antequera, 2007). However, regions of high CpG density are often unmethylated and located on active promoters (Bird, 1985; Larsen et al., 1992; Saxonov et al., 2006). In contrast, promoters with methylated CpG sites are repressed (Feinberg and Vogelstein, 1983; Bird, 2002; Jaenisch and Bird, 2003; Vaissière et al., 2008). Furthermore, DNA methylation is generally anti-correlated with CpG density (Lövkvist et al., 2016), i.e., in regions where CpG density is high, average methylation levels are low, and vice versa.

Nucleosomes form the building blocks of chromatin which in turn affect the accessibility of the DNA (Richmond and Davey, 2003; Henikoff and Smith, 2015). Reduction of accessibility might indeed compromise the access transcription factors have to the DNA and consequently alter the transcriptional state (Lorch et al., 1987; Schones et al., 2008; Shivaswamy et al., 2008; Portela and Esteller, 2010; Jin et al., 2011). This is in line with findings where active promoters are found to be depleted of nucleosomes and show well-positioned nucleosomes after the transcription start site (TSS) (Yuan et al., 2005; Jin and Felsenfeld, 2007; Bai and Morozov, 2010; Portela and Esteller, 2010; Bai et al., 2011; Jones, 2012; Kelly et al., 2012; Turner, 2014). In contrast, methylated CpG sites were found to be covered by nucleosomes and were observed to affect both stability and positioning of the nucleosomes (Pennings et al., 2005; Jin et al., 2011; Jones, 2012; Collings et al., 2013; Collings and Anderson, 2017; Nakamura et al., 2017). An increase in nucleosome occupancy due to DNA methylation was also found to correlate with deactivation of genes (Choy et al., 2010; Yazdi et al., 2015). Some studies indicate nucleosome occupancy to be dependent on CpG density (Tillo et al., 2010; Lee and Lee, 2011). Less CpG dense promoters tend to be methylated and covered by nucleosomes (Jones, 2012). Active unmethylated promoters would hence be highly accessible as they are free from nucleosomes (Kundaje et al., 2015).

Despite all these findings, causal links between nucleosome occupancy and CpG methylation are far from proven. One hypothesis is that methylated CpG sites promote nucleosome formation, which would be consistent with the finding that promoters with unmethylated CpG sites are nucleosome depleted. Other studies argue for a different explanation: there, nucleosomes promote the presence and patterning of methylation. Following this logic, nucleosome depleted regions would not target methylation and CpG sites there would consequently be free of methylation (Chodavarapu et al., 2010; Thurman et al., 2012; Taberlay et al., 2014). Statistical relationships between DNA methylation and nucleosome occupancy might even be brought about indirectly through factors that bind to methylated DNA and then affect nucleosome positioning (Segal and Widom, 2009). The effect of DNA methylation on gene expression is not completely clear. It is unknown whether DNA methylation inactivates genes or if gene silencing precedes DNA methylation (Jones, 2012). Methylation might be a secondary event and serve to imprint gene activity (Bird, 1985). However, a recent experiment has shown that methylated DNA can decrease gene expression (Vojta et al., 2016).

In this work, we probe possible causal links between DNA methylation and nucleosome occupancy by analyzing genomic regions that are not located on or near promoters. This rules out possible impact by transcription, primarily transcription initiation. Additionally, we compare promoters with different expression levels with regions outside promoters with similar CpG densities. Regions away from promoters expectedly lack the characteristic nucleosome patterns observed on promoters. We however find regions with similar CpG densities, on promoters or not, to have similar methylation levels—suggesting a clear link between methylation and CpG density, but eliminating any direct causal relation between nucleosome occupancy and CpG density or methylation. The patterns on promoters seem to be highly correlated with transcription. However, they are all independent of CpG density and DNA methylation.

## Materials and methods

CpG positions are retrieved from the reference genome hg18, downloaded from: hgdownload.cse.ucsc.edu/downloads.html. The position of any CpG site is defined as the position of the cytosine on the 5′-3′ strand. We included 18,015 promoters in the analysis and analyzed the regions around the transcription start site (TSS) from the −1,000 to +1,000 position (Hawkins et al., 2010). These promoters are primarily protein coding promoters. To investigate methylation levels and nucleosome occupancy in the human genome, we use data from the NOME-seq method (Kelly et al., 2012), which provides methylation data and nucleosome occupancy from the same read of IMR90 cells. The data is downloaded from NCBI Gene Expression Omnibus (GEO) (http://www.ncbi.nlm.nih.gov/geo/) under accession number GSM1001125. In the NOME-seq method, DNA is treated with M.CviPI that methylates the GpC nucleotides that are not covered by nucleosomes. The methylation status of CpG sites and the GpC nucleotides are then determined using the bisulphite conversion method. For each position the methylation information is represented as the number of methylated reads divided by the total number of reads. The nucleosome occupancy for each position is then obtained from 1−GpC methylation level.

We confirm that, as reported in previous studies (Bird, 1980), CpG sites are under-represented in the genome: there are 0.9% compared to the 3.4% that would be statistically expected from the C+G content, however, the GpC content corresponds to what is expected from the C+G content. Hence there are GpCs genome-wide to accurately measure the nucleosome occupancy using the NOME-seq method (Kelly et al., 2012).

**Figure 1:**
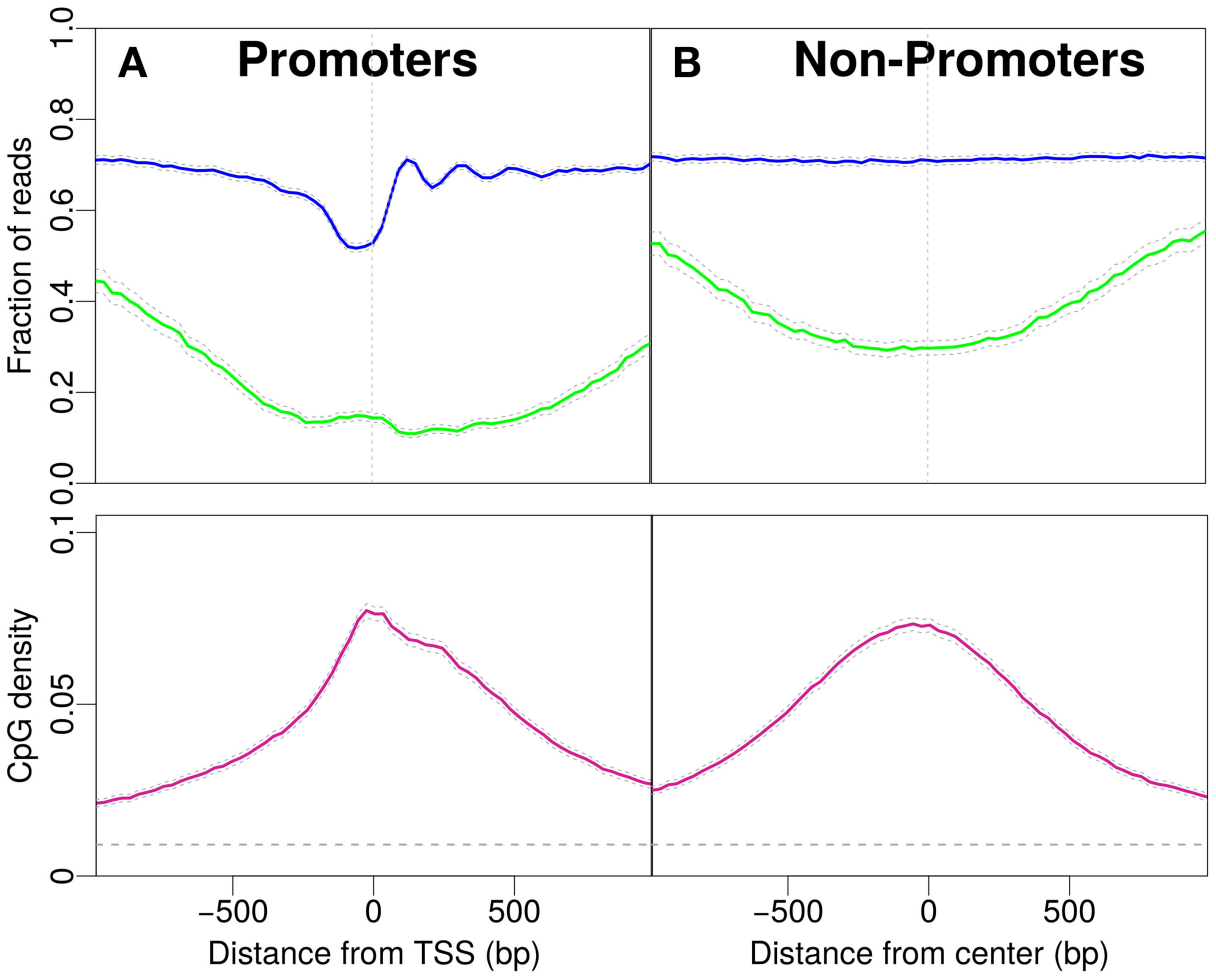
Profiles for nucleosome occupancy (blue), methylation level (green) and CpG density (pink). For each position in the region the nucleosome occupancy, methylation level and CpG density is averaged over all promoters (see Materials and Methods). **(A)** Promoter regions upstream and downstream the TSS (x-axis origin). **(B)** Analogous to **(A)** for non-promoter regions. The regions are chosen to resemble the CpG density profile in **(A)**. The gray dashed line indicates the average CpG density (0.009) in the genome. 18,015 promoters and 17,982 regions are analyzed in **(A,B)**.

CpG density, methylation level, and nucleosome occupancy are averaged in the vicinity of promoters. In practice, this means that for each position (measured in base pairs away from promoters, see e.g., Figure 1), the average number of nucleosomes and CpG sites is determined for all relevant promoter regions. For each position, nucleosome occupancy and methylation levels are averaged over promoters with measurements, the ones without measurements are disregarded in the averaging process. CpG densities are averaged over all promoters for each position unless a CpG position is already part of a promoter region. It is then disregarded in the averaging process to avoid double counting. For brevity, in the following we use the term *CpG density* to refer to the CpG site number density per base pair. All profiles are further averaged within moving windows of 30 *bp*.

**Figure 2:**
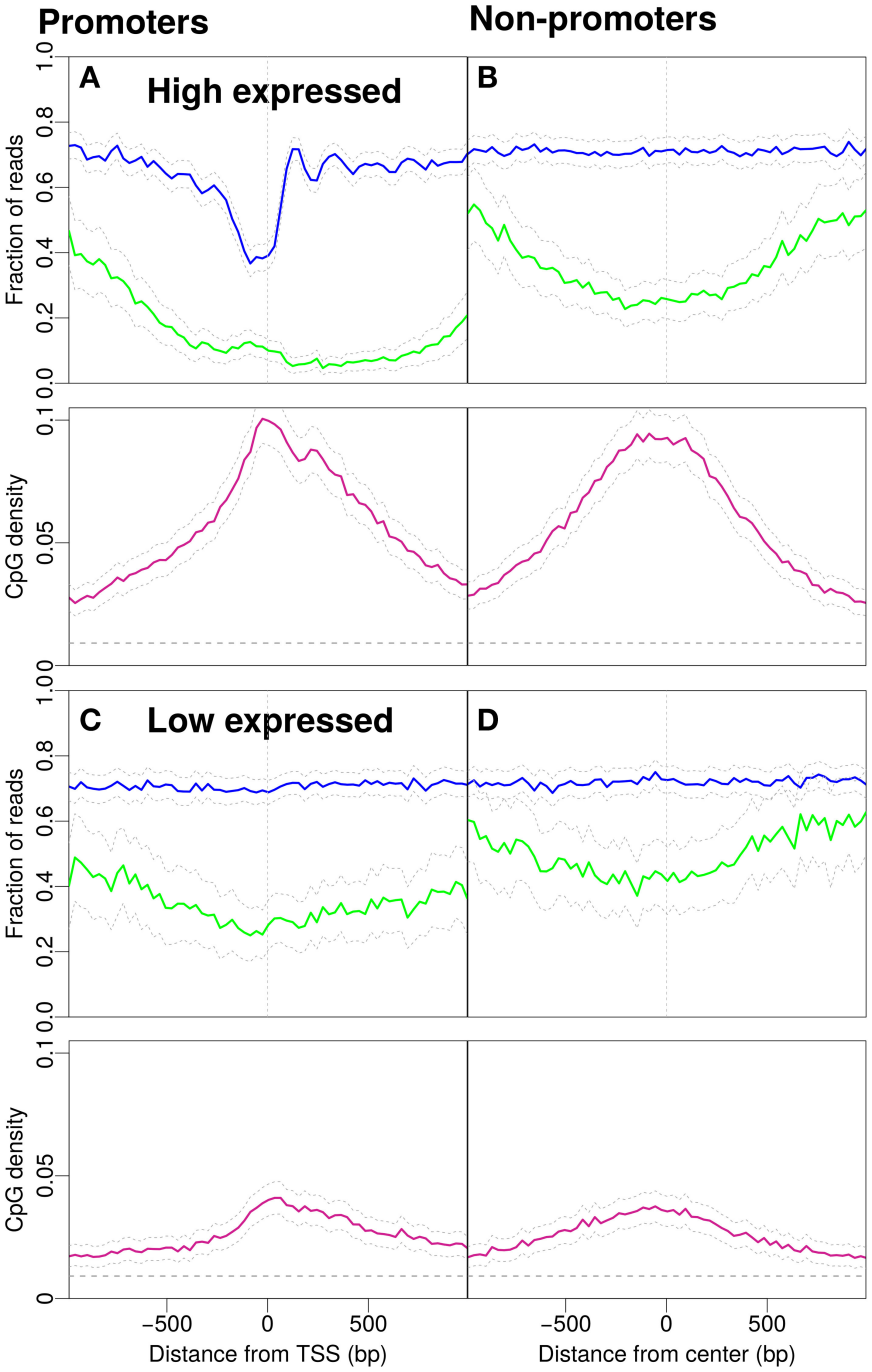
Nucleosome occupancy (blue), methylation level (green) and CpG density (pink). **(A)** Promoters with high gene expression (900 promoters selected, see Materials and Methods). **(B)** Non-promoter regions with similar CpG density profiles as in **(A)** (898 regions selected, see Materials and Methods). Methylation drops around the midpoint of the region and the GpCs are, on average covered by nucleosomes. **(C)** Profiles for promoters with low gene expression (901 promoters selected, see Materials and Methods). **(D)** Non-promoter regions with similar density profiles as in **(C)** (900 regions selected, see Materials and Methods). The gray dashed line indicates the average CpG density (0.009) in the genome.

In Figure 2, we distinguish expression levels for promoters. Figures 2A,C, respectively, show the 5% of promoters with highest and lowest expression levels. The expression levels were taken from the literature (Hawkins et al., 2010).

The data for the non-promoter regions (Figure 1B) are selected to resemble the density profile of the promoter regions. To do this, we pooled the rest of the genome for CpG clusters. A CpG cluster is a region of CpG sites with inter-CpG distances less than 20 *bp*. Furthermore, CpG clusters are grouped together if the distances between the clusters are less than 50 *bp*. Cluster regions of 1,000 *bp* on either side are defined around the center of the clusters and for each promoter, a cluster region with the same number of CpG sites as the promoter region is selected. The center of the region is shifted until the number of CpG sites on each side of the center is equal to the number of CpG sites on either side of the TSS of the promoter. The selected non-promoters are not overlapping regions within 1,000 *bp* on each side of the TSS of the promoters, i.e., locations for transcription initiation. Hence, the regions containing the promoter regions are specifically excluded from this selection process, in order to avoid statistical artifacts. It is not distinguished whether the non-promoters are within transcribed units (i.e., non-coding RNA) or not.

## Results

First, we focus on promoters and observe patterns of nucleosome occupancy, CpG density and methylation (see Materials and Methods). Before the transcription start site (TSS) we distinguish characteristic features with comparably low nucleosome occupancy, which we here refer to as “nucleosome decreased regions” (NDR). Note that we here distinguish these from “nucleosome depleted regions,” a term common in the literature, used for regions which are essentially free of nucleosomes. The region after the TSS shows well-positioned nucleosomes, which is reflected by an oscillatory pattern of the statistical occupation probability. In these promoter regions, the majority of CpG sites is unmethylated and the methylation level drops to a minimum of approximately 0.15 per CpG site close to the TSS (see Figure 1A). The CpG density near the TSS (~0.08) is one order of magnitude larger than the average CpG density in the entire genome (~0.009). The density profile peaks around the TSS and drops off away from the TSS (Figure 1A).

To compare the CpG density, methylation and nucleosome occupancy patterns within genomic regions away from promoters, we selected only non-promoter regions of similar length that produced similar CpG density profiles as in Figure 1A (see Materials and Methods). Indeed, the methylation level is also low in these regions, with a minimum near the center (Figure 1B). The drop in methylation level is again accompanied by a simultaneous increase in CpG density. The GpCs are, on average covered by nucleosomes and the nucleosome occupancy profile is constant. The characteristic patterns observed for IMR90 cells in Figure 1A and the corresponding profiles for non-promoter regions are also observed for glioblastoma cells (see Supplementary Material). When comparing the panels in Figures 1A,B, patterns in CpG density and methylation are qualitatively similar, but we do acknowledge a quantitative difference, especially in CpG methylation: While the average value of the methylation profile reaches as low as 0.1 per site for the promoter regions, away from promoters the minimum lies somewhat higher (~0.25). However, it is remarkable that the features in CpG density and methylation are so similar, despite the complete lack of feature in nucleosome occupancy for the non-promoter regions. These findings make it unlikely to suspect a direct, causal link between nucleosome occupancy and CpG density. While there may be some impact on CpG methylation, the continued presence of the dip in CpG methylation for the non-promoter regions hints toward a secondary role of nucleosome occupancy in driving CpG methylation.

What is the effect of gene expression levels? To this end, we investigate the patterns separately for promoters with high and low gene expression (Figure 2). Promoters with high gene expression have a more pronounced NDR and lower methylation compared to Figure 1A. The profile for CpG density increases around the TSS (Figure 2A). The non-promoter regions with a similar CpG density profile as the high-expression promoters also show a similar decrease in methylation around the center of the respective regions, however, the nucleosome occupancy profile is as expected constant away from promoters (Figure 2B). Low expression promoters have lower CpG densities and higher methylation levels. The promoters are occupied by nucleosomes but the positioning is no longer apparent, i.e., nucleosome occupancy is essentially constant as a function of position (Figure 2C). Non-promoters are selected to resemble the CpG density profiles of low-expression promoters. The methylation level is higher than that on promoters and the nucleosome occupancy remains constant and has similar magnitude as with the low-expression promoters (compare Figure 2C and Figure 2D).

**Figure 3:**
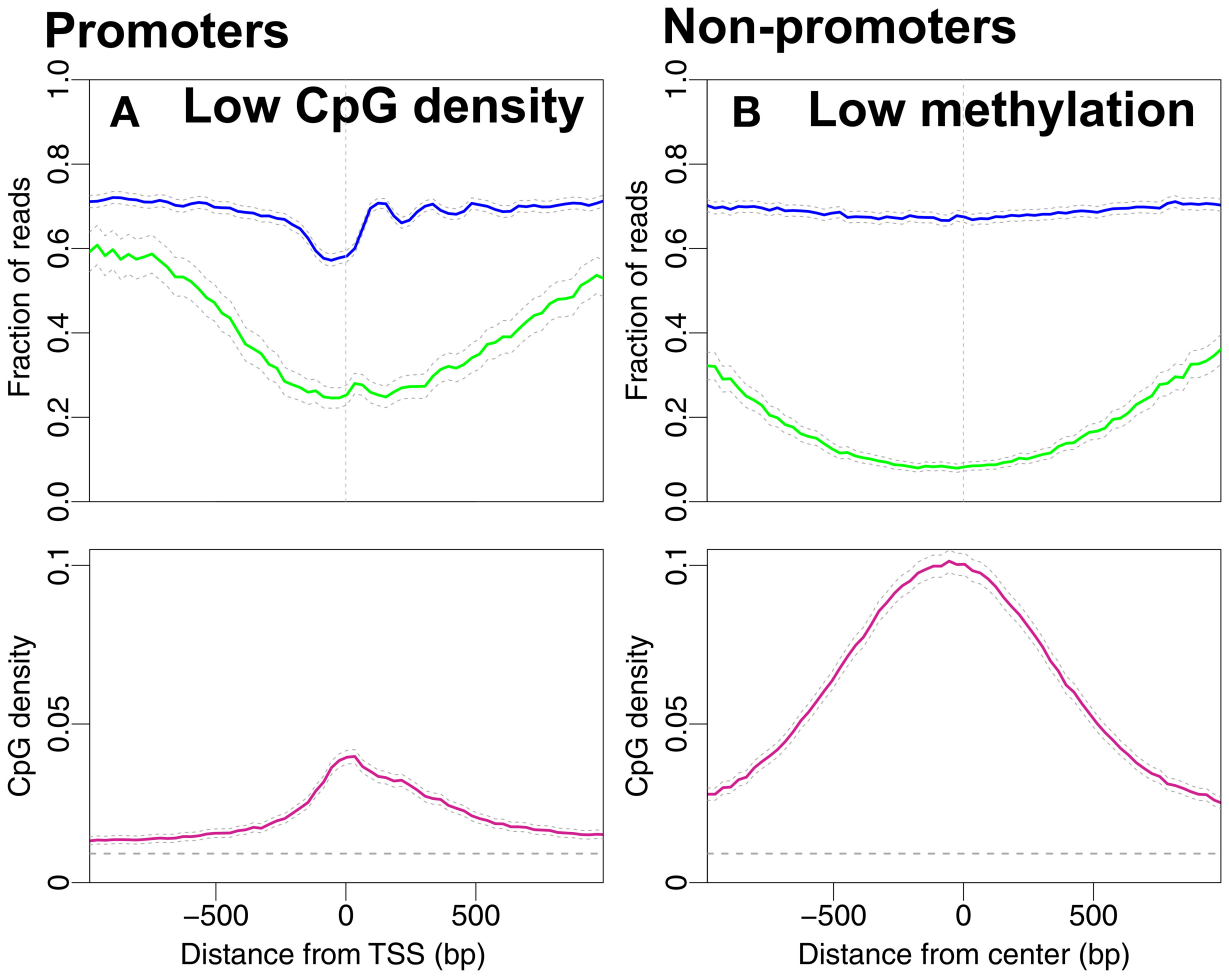
Nucleosome occupancy (blue), methylation level (green) and CpG density (pink). **(A)** Promoters with low CpG densities, resembling the density profile in Figure 2C. 8,215 promoters with average CpG density lower than the average CpG density among all 18,015 promoters in Figure 1A (≈0.04). **(B)** Regions with low methylation levels are chosen from the non-promoter regions in Figure 1B. 7,367 regions with average methylation lower than the average methylation level of all non-promoter regions in Figure 1B (≈0.39). The gray dashed line indicates the average CpG density (0.009) in the genome.

In Figure 3A we consider the reversed causality by selecting promoters with low CpG density. These are promoters with average CpG densities that are lower than the average density of all 18,015 promoters in Figure 1A. Of the 8,215 promoters selected, 230 are high-expressed promoters and 713 low-expressed promoters. It is plausible that the drop in methylation level around the TSS and the NDR followed by well-positioned nucleosomes are the profiles of the high-expressed promoters. To investigate if the nucleosome occupancy of non-promoter regions is affected by unmethylated CpG sites we select regions with low methylation. We define these as regions with average methylation level along the entire 2,001 *bp* region lower than 0.39 (the average methylation levels of all regions in Figure 1B). The CpG density profile reaches larger values with a peak around the TSS. The peak in CpG density is of similar magnitude as that of high-expression promoters (compare Figure 2A and Figure 3B). Interestingly, the corresponding CpG methylation level is now very similar for the two. Importantly, the nucleosome occupancy profile remains constant in Figure 3B, around the same level as in Figures 2B–D. This finding further adds to a picture where nucleosome occupancy varies systematically within promoter regions, but its effect on CpG density or methylation is less clear.

From the promoter profiles (Figures 1A, 2A,C) we confirm that the NDR is more pronounced in highly expressed genes. In addition to high CpG density, also gene expression correlates with reduced methylation level. However, if we consider regions that are not exposed to transcription (Figures 1B, 2B,D) we observe no change in nucleosome occupancy when CpG density or methylation change. Additionally, typical characteristics of active promoters are observed in Figure 3A despite overall low CpG density. Dynamical models imply that methylation level may be influenced by changes in CpG density (Haerter et al., 2014; Lövkvist et al., 2016), which is in line with the correlations see in our data analysis here (Figures 2B,D). When unmethylated regions are selected, those regions have high CpG densities (Figure 3B). Thus, CpG density and methylation are dependent and are anti-correlated, with little additional influence by nucleosome occupancy.

**Figure 4:**
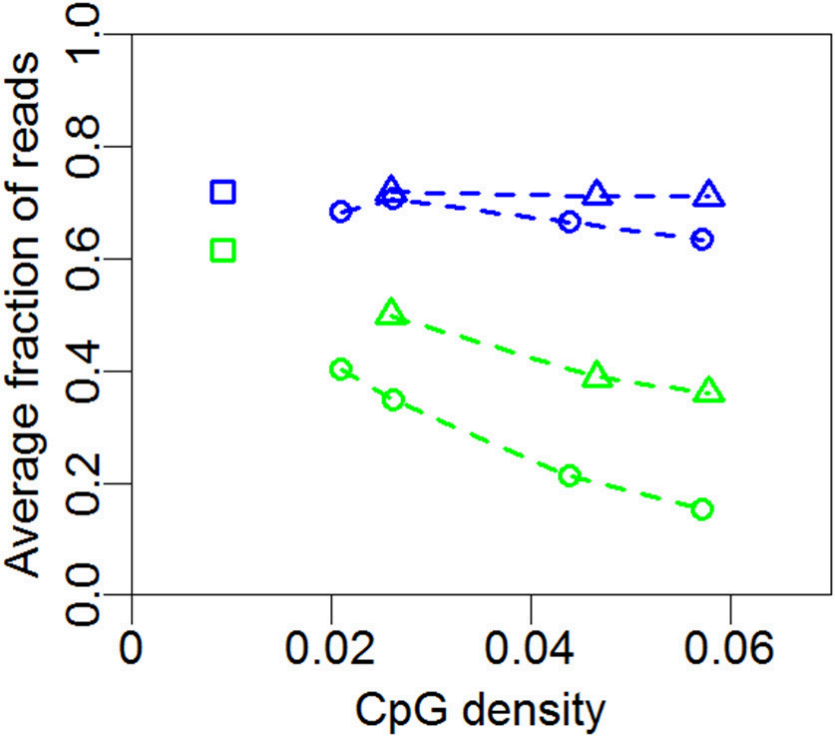
Mean nucleosome occupancy and mean methylation. Mean methylation (green) and mean nucleosome occupancy (blue) of the profiles in Figures 1, 2 and 3A for promoter (circles) and non-promoter regions (triangles). Each point represents the mean methylation or nucleosome occupancy of a given profile in Figures 1, 2 or 3A. Squares represent whole-genome averages.

In Figure 4 we summarize the profiles of the regions in Figures 1, 2. For each profile, the mean nucleosome occupancy and mean methylation is shown for the corresponding mean of CpG density. The methylation and nucleosome occupancy is hence shown as a function of CpG density. Here it is also observed that methylation is decreasing with CpG density and nucleosome occupancy remains constant. The methylation level is higher for non-promoter regions but follows the same decrease in methylation with increasing CpG density. Together, these findings suggest that nucleosome occupancy is correlated with expression level rather than CpG density or methylation level.

## Discussion

What determines nucleosome and DNA methylation patterns on promoters and their impact on gene activation or silencing? In this work, we have analyzed typical nucleosome patterns on active promoters, a nucleosome decreased region (NDR) followed by well-positioned nucleosomes. The methylation level drops around the TSS, a feature that is accompanied by simultaneously increasing CpG density. We have also confirmed that less expressed promoters are occupied by nucleosomes with higher methylation levels. Apart from these known features, less expressed promoters have lower densities of CpG sites.

We however make two observations: when selecting promoters with low CpG density and high methylation, NDRs and well-positioned nucleosomes are still observed; non-promoter regions with similar CpG density profiles as promoters have nucleosome occupancy that is indistinguishable from random genomic regions. These findings make transcription, not CpG density or DNA methylation, the strong candidate for causing nucleosome decreased regions.

DNA methylation and CpG density, on the other hand, are correlated, irrespective of whether the regions are located on promoters or not. For comparable CpG density profiles, methylation levels on promoters are slightly lower than those away from promoters—indicating that the methylation level may be somewhat influenced by gene expression. However, CpG density remains the main predictor of methylation level.

There are many ways in which nucleosomes states interfere with DNA methylation (Kobor and Lorincz, 2009; Chodavarapu et al., 2010; Jones, 2012), and it is hence likely that also the occupation of a certain region by nucleosomes influences maintenance or removal of DNA methylation. Our results indicate that a reduction of nucleosome occupancy is not sufficient to lead to strong shifts in the DNA methylation level, however, the complex relationship between nucleosome occupancy and DNA methylation should be tested further. Ideally, experimental studies should attempt measurements that can simultaneously access DNA methylation and the nucleosome states at a similar location where the CpG densities could be engineered.

We have here focused on the contrast between promoter regions, i.e., locations near transcription start sites, and non-promoter regions. However, we have left unanswered whether there might also be an influence of nucleosome occupancy on DNA methylation in regulatory elements, e.g., enhancers and insulators. Enhancers are CpG poor with intermediate methylation levels and nucleosome occupancy (Lister et al., 2009; Jones, 2012; Kundaje et al., 2015). Furthermore, it is important to characterize this interrelation on exons compared to introns: whereas methylation around the transcription start site is found to inhibit transcription, methylation of other parts of the gene body is found not to block transcription (Jones, 2012). Exons are found to be more methylated and occupied by more nucleosomes compared to introns (Chodavarapu et al., 2010; Jin et al., 2011). The non-promoter regions, as we define them here, are regions that do not overlap with promoters, but their relatively low methylation level and high CpG density could point to a—so far unknown—regulatory function in the genome.

In this work, we do not rule out a possible interrelation between DNA methylation and histone modifications on the nucleosomes. While we propose that there is no strong effect of nucleosome occupancy on DNA methylation, the histone modifications of the nucleosomes nonetheless are implicated in recruitment of methylation: Histone methylases interact with DNA methylases and—as a result—methylated histones can indeed promote DNA methylation (Fuks et al., 2003; Cedar and Bergman, 2009). We suggest that the variations in nucleosome occupancy discussed here may not be strong enough to decisively influence DNA methylation levels—possibly, the DNA methylation-nucleosome complex is robust enough to ensure cooperation between these two systems, even if nucleosome occupancy is reduced. Additionally, there might be other factors keeping regions with high CpG density free from methylation. An example is Tet1, a member of a family of proteins that contain a CXXC zinc finger domain, that keeps regions with high CpG densities unmethylated (Tahiliani et al., 2009; Deaton and Bird, 2011).

The data used in the current study are static and therefore do not contain information about methylation levels and nucleosome occupancy over time. Which features may change when CpG sites switch methylation state or when nucleosomes reposition along the genome? In addition, there are differences in methylation between different cell and tissue types (Lister et al., 2009; Kundaje et al., 2015). Moreover, in (Lövkvist et al., 2016) we observed the anti-correlation between CpG density and methylation in different cell types. Additionally, we observed qualitatively similar results between IMR90 cells and glioblastoma cells (See Supplementary Material).

This paper has addressed the basic interplay between three players involved in epigenetic states: nucleosome occupancy, CpG site density, and DNA methylation. Our findings suggest that DNA methylation is predominantly influenced by the density of CpG sites and only to a weaker extent by fluctuations in nucleosome presence. We suggest experiments where formerly CpG dense and methylation poor promoter regions are engineered to accommodate fewer CpG sites. Studying the resulting methylation could help uncover the direct interplay between nucleosomes, CpG sites and the level of methylation in a controlled experimental set-up.

## Author contributions

CL performed all data analysis. CL, KS, and JOH wrote and edited the manuscript. CL, KS, and JOH read and approved the final manuscript.

### Conflict of interest statement

The authors declare that the research was conducted in the absence of any commercial or financial relationships that could be construed as a potential conflict of interest.
