# Supplementary material for: Exploring the Link between Nucleosome Occupancy and DNA Methylation
Source: Front Genet. 2018 Jan 12;8:232. doi: 10.3389/fgene.2017.00232 (PMC5771128; doi:10.3389/fgene.2017.00232)
Supplement: Supplementary file 1 [file Presentation1.PDF]

Supplementary Material

Exploring the link between nucleosome occupancy and DNA methylation

Cecilia Lövkvist [1], **Kim Sneppen** [1] **and Jan O. Haerter** [1]

[1] *Center for Models of Life, Niels Bohr Institute, University of Copenhagen, Blegdamsvej 17, Copenhagen, Denmark*

Correspondence*:
Cecilia Lövkvist
ceclov@nbi.ku.dk

To investigate if the results in the main text are specific to IMR90 cells or not, we analyze additional data from glioblastoma cells (GBM Cells) from Kelly et al. (2012). The data is downloaded from NCBI Gene Expression Omnibus (GEO) (`http://www.ncbi.nlm.nih.gov/geo/`) under accession number GSM1001126 and GSM1001127. The data sets are mapped to the reference genome hg19 and to compare to the promoter regions in the main text, we select the refGene promoters (downloaded from `http://genome.ucsc.edu`) that overlap with the promoters in Hawkins et al. (2010).

In Figure 1A the nucleosome occupancy, methylation level, and CpG density are shown for GBM cells (GBM #157). The CpG density increases around the TSS simultaneously as the methylation levels decreases. We distinguish a NDR around the TSS as observed in the nucleosome occupancy profile of the IMR90 cells. Qualitatively the three profiles are similar to the profiles for the IMR90 cells in the main text. In Figure 2A we observe similar methylation, nucleosome occupancy and CpG density profiles for a second data set of GBM cells (GBM #248) as for the GBM #157 cells. For both data sets, we analyze non-promoter regions (1B and 2B) and we observe the methylation levels to decrease with increasing CpG density and the nucleosome occupancy profiles to be constant.

In Kelly et al. (2012) the nucleosome occupancy and methylation levels for the glioblastoma cells are already observed to be qualitatively similar to the IMR90 cells. We also observe similar patterns as observed for IMR90 cells in Figure 1A and 2A. Additionally, we also observe an increasing CpG density around the TSS and the non-promoter profiles show a constant nucleosome occupancy and decreasing methylation level with increasing CpG density. From this, we conclude that the features observed in IMR90 are also observed in glioblastoma cells and that these observations are generic for the three cell types.

REFERENCES

Hawkins, R. D., Hon, G. C., Lee, L. K., Ngo, Q., Lister, R., Pelizzola, M., et al. (2010). Distinct epigenomic landscapes of pluripotent and lineage-committed human cells. *Cell stem cell* 6, 479–491

Kelly, T. K., Liu, Y., Lay, F. D., Liang, G., Berman, B. P., and Jones, P. A. (2012). Genome-wide mapping of nucleosome positioning and dna methylation within individual dna molecules. *Genome research* 22, 2497–2506

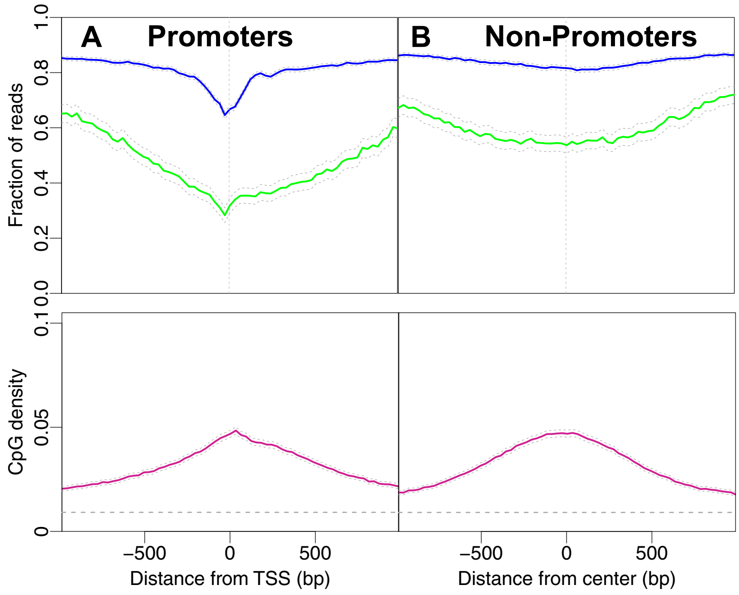

Figure 1. Profiles for nucleosome occupancy (blue), methylation level (green) and CpG density (pink) for GBM #157 cells. For each position in the region the nucleosome occupancy, methylation level and CpG density is averaged over all promoters (see Methods). (A) regions upstream and downstream the TSS (x-axis origin). (B) to (A) for non-promoter regions. The regions are chosen to resemble the CpG density profile in (A). The gray dashed line indicates the average CpG density (0.009) in the genome. 15683 promoters and 15674 regions are analyzed in (A) and (B).

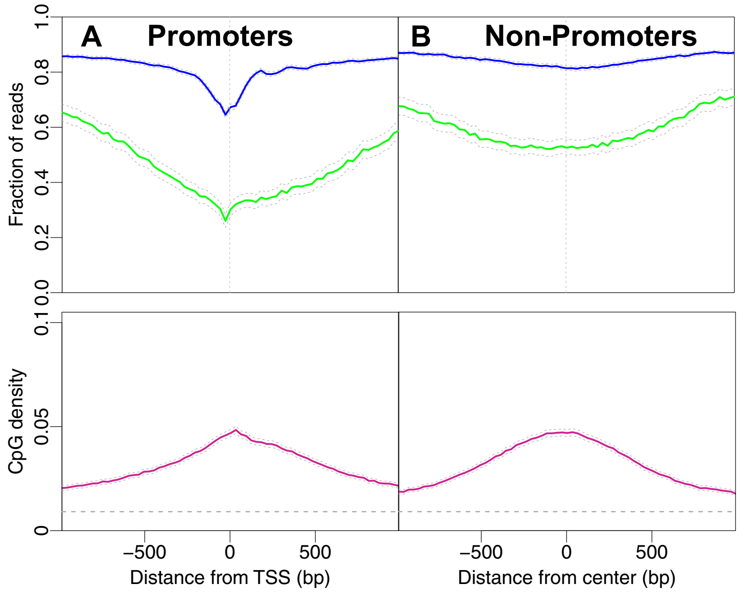

Figure 2. Profiles for nucleosome occupancy (blue), methylation level (green) and CpG density (pink) for GBM #248 cells. For each position in the region the nucleosome occupancy, methylation level and CpG density is averaged over all promoters (see Methods). (A) regions upstream and downstream the TSS (x-axis origin). (B) Analogous to (A) for non-promoter regions. The regions are chosen to resemble the CpG density profile in (A). The gray dashed line indicates the average CpG density (0.009) in the genome. 15683 promoters and 15674 regions are analyzed in (A) and (B).
